# Self-reported diet management, dietary quality, and blood pressure control in Korean adults with hypertension

**DOI:** 10.1186/s40885-019-0130-z

**Published:** 2019-12-15

**Authors:** Jee-Seon Shim, Sun Jae Jung, Hyeon Chang Kim

**Affiliations:** 1 Department of Preventive Medicine, Yonsei University College of Medicine, Yonse-ro 50-1, Seodaemun-gu, 03722 Seoul, Republic of Korea; 2 Cardiovascular and Metabolic Diseases Etiology Research Center, Yonsei University College of Medicine, Yonse-ro 50-1, Seodaemun-gu, 03722 Seoul, Republic of Korea; 3 Department of Epidemiology, Harvard T.H. Chan School of Public Health, 677 Huntington Ave. #505, Boston, MA 02115 USA

**Keywords:** Hypertension, Blood pressure, Diet therapy, Self-management, Control

## Abstract

**Objectives:**

Hypertension control is a major public health concern. Daily preventive practices of the affected individual are essential for controlling blood pressure (BP). We investigated the association of diet management practice, dietary quality, and BP control among Korean adults with known hypertension.

**Methods:**

We included 4107 participants aged 40–79 years who reported physician-diagnosed hypertension in the Korea National Health and Nutrition Examination Survey 2013–2016. Dietary management practice was defined by self-report, and dietary intakes were assessed by a 24-h dietary recall. Dietary quality and adherence were evaluated based on the Korean Healthy Eating Index (KHEI) using food and nutrient intakes assessed by a 24-h dietary recall. BP control was defined as systolic/diastolic BP < 140/90 mmHg.

**Results:**

While the prevalence of dietary management was higher in women than men, BP control rate was not different by sex. Dietary management practice had no significant association with BP control in both men and women. Only in men, dietary quality was positively associated with BP control (OR: 1.10 per KHEI 10 score increase, 95% CI: 1.00–1.20, *p*-value = 0.04). Men who had a highly adherent diet seemed to have a higher possibility of BP control, but there was no statistical significance (OR: 1.54, 95% CI: 0.84–2.81, *p*-value = 0.16).

**Conclusions:**

A high-quality diet was positively associated with BP control in Korean men aware of their hypertension. Our findings highlight the beneficial impact of dietary management as a means for achieving blood pressure control.

## Background

Hypertension is a global health problem. The prevalence of hypertension worldwide was 26% in 2000, and the total number of adults with hypertension will reach an estimated 1.56 billion in 2025 [1]. Elevated blood pressure (BP) is a leading cause of cardiovascular diseases, disability, and premature death [2, 3]. Along with population ageing, the burden of hypertension on our societies is predicted to increase continuously. However, hypertension is modifiable [4–7]; and well-controlled BP can prevent clinical complications, enhance quality of life, and improve prognosis [8–11].

The optimal BP for adults with hypertension can be achieved through pharmacological treatment and nonpharmacological interventions. Daily preventive practices of the affected individual are essential for BP control [12]. During the past decades, the hypertension treatment rate has increased remarkably, especially in some countries including South Korea; and the BP control rate among hypertensive adults treated with medication has also substantially improved [13, 14]. The reason for this may be that self-management of medication has become easier with alleviation of potential barriers surrounding pharmacological treatment, i.e., limited health care, drug expense, inconvenience of dosing, and severity of side effects [15]. However, recent indices of hypertension management have remained stable; and a considerable number of medicated adults with hypertension do not reach the optimal BP control level [14].

Lifestyle modifications such as healthy diet, low sodium consumption, sufficient physical activity, and limited alcohol consumption have been strongly recommended for all hypertensive individuals. These modifications can serve as an initial treatment before the start of medication or as an adjunct to medication in persons already on pharmacological treatment [16, 17]. There are few studies focusing on hypertensive individuals’ lifestyle management practices and their effects on BP control. According to some previous studies, adherence to lifestyle modification, particularly dietary modification, is much lower than that of medication compliance [18, 19]. This is true despite the noted beneficial impact of lifestyle modification in many clinical trials and epidemiologic studies. Contrary to pharmacological treatment, lifestyle modification has few or no harmful side effects and leads to improved overall cardiovascular health in addition to BP reduction [5]. Lifestyle modification can reduce the requirement for BP-lowering drugs [6]. Therefore, a more aggressive movement from pharmacological treatment toward lifestyle modification seems necessary.

To the best of our knowledge, there are few studies that evaluate dietary management practices and their effects on BP control among adults diagnosed with hypertension. Thus, we aimed to investigate the association of self-reported dietary management, dietary quality, and BP control among Korean adults with known hypertension.

## Methods

### Data source and study population

We used the data from the Korea National Health and Nutrition Examination Survey (KNHANES) 2013–2016. KNHANES is a nationwide survey designed to assess the health and nutritional status of Koreans. Annually, 10,000 individuals aged 1 year or over are recruited as representative samples, and the response rate is nearly 80%. A considerable quantity of information on anthropometric measures, disease-related profiles, health-related behaviors, and dietary intake and behavior is collected through three component surveys comprised of a health interview, health examination, and nutrition survey. KNHANES not only provides health-related statistics in Korea, but also has been widely used for its abundant research data. More details regarding KNHANES have been described elsewhere [20].

Among all participants aged 40–79 years (*n* = 16,334), we excluded those who had insufficient data (*n* = 3356), those who were pregnant or breastfeeding (*n* = 24), and those who had not yet been diagnosed with hypertension (*n* = 8847). Thus, a total of 4107 adults who reported to have been diagnosed with hypertension by a physician were included in this study.

### Dietary management and dietary quality

The nutrition survey includes information regarding diet management behavior as well as information on dietary intake. During the survey, each participant was asked to answer the question “*Are you managing your diet for any special reason?*” and, if yes, to choose the main reason for dietary management among three possible reasons of disease, weight control, or other. We defined the presence of dietary management using the self-report answer and divided participants into diet-managing or non-managing groups.

Participants’ food and nutrient intakes were assessed by a 24-h dietary recall [20]. Dietary quality was evaluated based on the Korean Healthy Eating Index (KHEI) using information on dietary intakes. The KHEI, a measure of overall diet quality as specified by the key dietary recommendations and 2010 Dietary Reference Intakes of Koreans (2010 KDRIs), was developed to assess comprehensive diet quality of Korean adults [21, 22]. The KHEI includes a total of 14 components: 8 items recommended for adequate food consumption (breakfast, mixed grains, total fruits, fresh fruits excluding fruit juice, total vegetables, vegetables excluding Kimchi and pickles, meat/fish/eggs and beans, and milk and milk products); 3 items for moderate consumption (saturated fatty acids, sodium, and sweets and beverages); and 3 items for balanced consumption (carbohydrates, total fat, and energy). The score for each item ranges from 0 to 5 (or 10), and the maximum score is 100. A higher score means a more healthy diet.

We also evaluated dietary adherence based on KHEI score and dietary sodium intake as follows: ‘highly adherent’ (both low sodium intake (≤ 2400 mg/day) and a high quality diet (highest quartile of KHEI score, Q4); ‘slightly adherent’ (either low sodium intake or a high quality diet); and ‘non-adherent’ (both high sodium intake (> 2400 mg/day) and a low quality diet (low quartiles of KHEI score: Q1–3).

### Blood pressure control

Trained researchers measured BP according to a predefined protocol [20]. After a rest of at least 5 min in a sitting position, BP was measured three times at 30 s intervals. The mean of the latter two systolic and diastolic BP measurements was used as participant BP. BP control was defined when the systolic and diastolic BP status met a target level < 140 and < 90 mmHg, respectively.

### Ethical aspects

The KNHANES protocols were approved by the Institutional Review Board (IRB) of KCDC (2013-07CON-03-4C, 2013–12-EXP-03-5C). All participants provided written informed consent. The study procedures were in accordance with the ethical standards of the responsible institutional committee on human experimentation and were in accordance with the Helsinki Declaration (of 1975 as revised in 2008).

### Statistical analyses

Demographic and disease-related data were presented as mean ± SD or as frequency (%). The differences of mean and distribution were tested using t-tests and chi-square tests. The associations between dietary management and BP control were examined using logistic regression analysis, and the ORs and 95% CIs are presented. We adjusted for potential confounding factors of age (y, continuous); duration of hypertension (y, continuous); cardiovascular comorbidities (yes or no) such as stroke, myocardial infarction, angina pectoris, diabetes, and dyslipidemia; family history of hypertension (yes or no); obesity (≥25.0 kg/m^2^; < 25.0 kg/m^2^); smoking (current smoking, non-, or past smoking); alcohol consumption (drinking ≥1 unit/month during the previous year; non-drinking or drinking < 1 unit/month); walking (≥5 days/week and ≥ 30 min/day; non-walking or less than 5 days/week or < 30 min/day); and BP-lowering medication (yes: taking a BP-lowering drug for more than 20 days/month; no: taking a BP-lowering drug less frequently or not at all). We also added HEI score (per 10 scores) and sodium intake (100 mg/day) as mutually exclusively plus the aforementioned variables in the analyses of the association of dietary quality, sodium intake, and BP control. Analyses were performed for all adults aware of their hypertension status, and the same analyses were performed for subgroups divided by presence of BP-lowering medication. All results were presented separately by sex. Additionally, sex- and age-stratified analyses for BP control were performed; and linear trends across dietary adherence and management were tested. All analyses were performed using the statistical software package SAS (version 9.4; SAS institute, Cary, NC, USA). *P*-values < 0.05 were considered statistically significant.

## Results

### Characteristics of adults with known hypertension

Among adults aware of their hypertension (*n* = 4107), 56.7% were women (Table 1). The hypertension duration was 9.6 ± 7.6 years, and 91.9% of patients were on BP-lowering medication. Cardiovascular risk factors such as obesity (49.2%), dyslipidemia (38.1%), diabetes (25.5%), and family history of hypertension (48.3%) were prevalent; and more than one in ten had a past history of cardiovascular disease such as stroke, myocardial infarction, or angina pectoris. Although all participants knew their hypertension status, only one-third were managing their diet.

**Table 1:** Demographic and dietary characteristics according to self-reported dietary management among adults with known hypertension

	Men			Women			Total
	Non-managing (*n* = 1329)	Managing (*n* = 450)	*p*-value	Non-managing (*n* = 1626)	Managing (*n* = 702)	*p*-value	
Age (year)	64.3 ± 9.5	64.4 ± 9.6	0.90	66.1 ± 9.0	64.0 ± 9.1	< 0.01	65.0 ± 9.3
40–64	611 (46.0)	204 (45.3)	0.86	631 (38.8)	333 (47.4)	< 0.01	1779 (43.2)
65–79	718 (54.0)	246 (54.7)		995 (61.2)	369 (52.6)		2328 (56.7)
Income (highest quartile)	322 (24.4)	126 (28.1)	0.23	368 (22.7)	182 (26.0)	0.05	998 (24.4)
Hypertension duration (year)	8.8 ± 7.7	11.1 ± 8.9	< 0.01	9.6 ± 7.8	9.9 ± 7.9	0.47	9.6 ± 7.9
Antihypertensive drug treatment (yes)	1197 (90.1)	409 (90.9)	0.68	1526 (93.9)	644 (91.7)	0.08	3776 (91.9)
Past history (yes)							
Stroke, myocardial infarction, angina pectoris	176 (13.5)	82 (18.3)	0.02	204 (12.7)	96 (13.9)	0.49	558 (13.8)
Diabetes	292 (22.0)	189 (42.0)	< 0.01	305 (18.8)	259 (37.0)	< 0.01	1045 (25.5)
Dyslipidemia	380 (28.6)	168 (37.3)	< 0.01	668 (41.1)	349 (49.7)	< 0.01	1565 (38.1)
Family history of hypertension (yes)	558 (42.0)	216 (48.0)	0.03	829 (51.0)	381 (54.3)	0.16	1984 (48.3)
Obesity (BMI ≥ 25.0 kg/m^2^)	615 (46.4)	234 (52.0)	0.04	797 (49.1)	373 (53.2)	0.07	2019 (49.2)
Current smoking	384 (29.5)	82 (18.6)	< 0.01	63 (4.0)	14 (2.0)	0.02	543 (13.5)
Drinking (≥1 unit per month)	919 (70.5)	262 (59.1)	< 0.01	398 (25.1)	193 (27.7)	0.21	1772 (44.0)
Walking (≥5 days/week and ≥ 30 min/day)	497 (38.5)	204 (46.0)	< 0.01	531 (33.6)	261 (37.9)	0.05	1493 (37.3)
Reasons for dietary management							
Diseases including hypertension	–	247 (54.9)		–	374 (53.4)		621 (54.0)
Weight control	–	176 (39.1)		–	303 (43.2)		479 (41.6)
Others such as dyspepsia	–	27 (6.0)		–	24 (3.4)		51 (4.4)
Dietary quality (KHEI score)^a^	61.9 ± 12.4	65.3 ± 12.4	< 0.01	65.5 ± 12.0	68.5 ± 12.1	< 0.01	64.8 ± 12.4
Dietary sodium intake	4167 ± 2614	4133 ± 3604	0.85	2875 ± 2117	2662 ± 1742	0.01	3395 ± 2525
Dietary adherence for hypertension^b^							
Non-adherent	808 (60.8)	234 (52.0)	< 0.01	585 (36.0)	191 (27.2)	< 0.01	1818 (44.3)
Slightly adherent	477 (35.9)	184 (40.9)		854 (52.5)	397 (56.6)		1912 (46.6)
Highly adherent	44 (3.3)	32 (7.1)		187 (11.5)	114 (27.2)		377 (9.2)

Values are means ± SDs or n (%)

^a^Dietary quality was assessed using KHEI (Korean Healthy Eating Index) and divided into low quality (Q1~Q3 of HEI score) and high quality (Q4 of HEI score)

^b^Dietary adherence was divided into ‘non-adherent’ (> 2400 mg sodium intake and low quartiles (Q1-Q3) of KHEI score), ‘slightly adherent’ (either ≤2400 mg sodium intake or highest quartile (Q4) of KHEI score), and ‘highly adherent’ (≤ 2400 mg sodium intake and highest quartile (Q4) of KHEI score)

Associated factors with dietary management differed by sex. In men, cardiovascular risk factors of longer hypertension duration, comorbid disease, family history of hypertension, and obesity were significantly associated with dietary management. Contrary to this, such positive associations seemed to be diluted in women. However, in both men and women, diet-managing adults had significantly higher KHEI scores, better dietary adherence, and, in women, lower sodium intake (although the mean sodium intake was higher than the recommended amount for hypertensive patients, 2400 mg/day).

### Self-reported dietary management and blood pressure control

Table 2 presents the odds ratio of self-reported dietary management for having controlled BP. The observed association differed by sex. After adjustment for potential confounding variables, diet-managing men had a higher OR for BP control than non-managing men (OR: 1.27, 95% CI: 0.98–1.66). This tendency remained unchanged in additional analyses for men with BP-lowering medication (OR: 1.28, 95% CI: 0.97–1.69). However, these associations did not reach statistical significance (*p*-value = 0.07 and 0.09, respectively). In women, no positive association of dietary management was found.

**Table 2:** Odds ratio of self-reported dietary management for blood pressure control among adults with known hypertension

	Non-managing		Managing		OR (95% CI) of dietary management for BP control		
	No. of Total	No. of controlled (%)	No. of Total	No. of controlled (%)	Model 1^a^	Model 2^b^	Model 3^c^
All adults with known hypertension							
Men	1329	936 (70.4)	450	338 (75.1)	1.27 (0.99, 1.62)	1.24 (0.96, 1.59)	1.27 (0.98, 1.66)
Women	1626	1153 (70.9)	702	485 (69.1)	0.89 (0.74, 1.09)	0.91 (0.75, 1.12)	0.90 (0.74, 1.11)
Hypertensive adults with antihypertensive drug treatment							
Men	1197	874 (73.0)	409	316 (77.3)	1.02 (0.96, 1.63)	1.28 (0.97, 1.68)	1.28 (0.97, 1.69)
Women	1526	1088 (71.3)	644	453 (70.3)	0.92 (0.75, 1.13)	0.96 (0.78, 1.18)	0.94 (0.76, 1.16)
Hypertensive adults without antihypertensive drug treatment							
Men	132	62 (47.0)	41	22 (53.7)	1.35 (0.67, 2.75)	1.23 (0.59, 2.59)	1.76 (0.77, 4.02)
Women	100	65 (65.0)	58	32 (55.2)	0.67 (0.35, 1.30)	0.58 (0.29, 1.17)	0.58 (0.28, 1.21)

^a^Adjusted for age

^b^Adjusted for duration of hypertension, comorbid status of cardiometabolic diseases such as stroke, myocardial infarction, angina pectoris, diabetes, or dyslipidemia, and family history of hypertension plus variables in the model 1

^c^Adjusted for obesity, smoking, drinking, walking, and antihypertensive drug treatment plus variables in the model 2

**Fig. 1:**
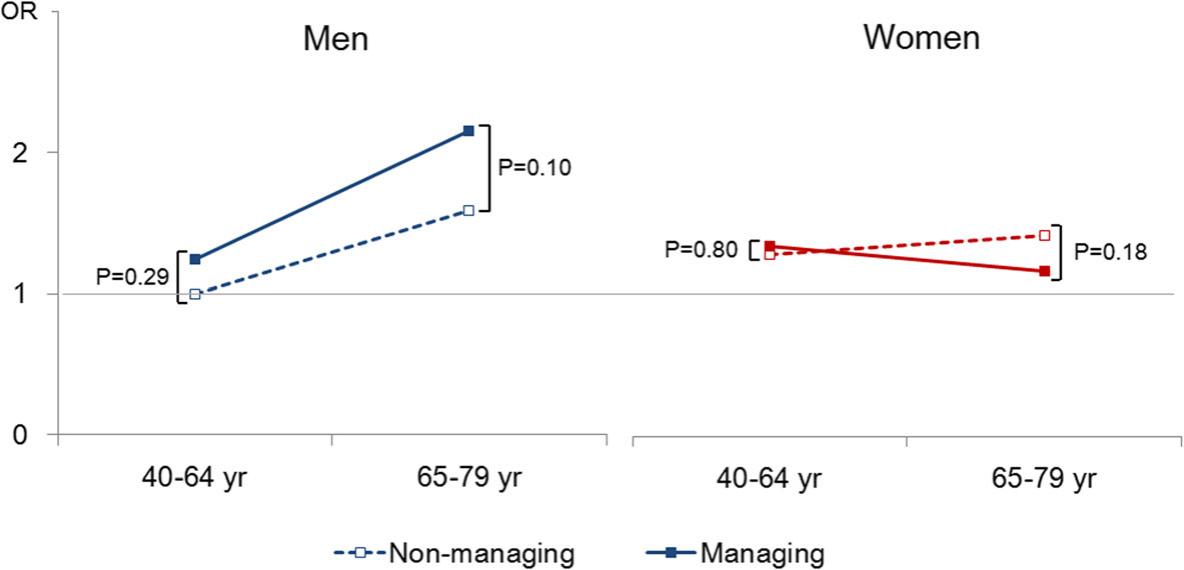
Odds ratio of self-reported dietary management for blood pressure control by sex and age group among adults with known hypertension^1^. ^1^Odds ratio was adjusted for age, duration of hypertension, comorbid status of cardiometabolic diseases such as stroke, myocardial infarction, angina pectoris, diabetes, or dyslipidemia, and family history of hypertension, obesity, smoking, drinking, walking, and antihypertensive drug treatment. ** < 0.01 of *p*-value for OR for blood pressure control compared with not-managing adults aged 40–64 years

Figure 1 shows the ORs for BP control according to sex and age group. Compared to middle-aged non-managing men, diet-managing older men had a significantly higher possibility for BP control (OR: 2.15, 95% CI: 1.41–3.27, *p*-value< 0.01), but there were no significant findings in women. We also analyzed the association of dietary management and BP control in each of the 4 groups divided by sex and age. The biggest association was observed in men ages 65–79 years old, but the association still did not reach statistical significance (OR 1.36, 95% CI: 0.94–1.98, *p*-value = 0.10). No beneficial association of dietary management and BP control was found in women.

### Dietary quality, sodium intake, and adherence for hypertension and blood pressure control

The associations of dietary quality and daily sodium intake with BP control are shown in Table 3. Higher KHEI score had a higher possibility for BP control, which was significant only in men (OR: 1.10, 95% CI: 1.01–1.20, *p*-value = 0.04). However, no negative association of higher sodium intake (per 100 mg increase) with BP control was found in men or women.

**Table 3:** Odds ratio of dietary quality, sodium intake, and adherence for blood pressure control among adults with known hypertension

	ORs for blood pressure control	
	Men	Women
All adults with known hypertension (*n* = 4107)		
N (total)/% of blood pressure control	1779/71.6	2328/70.4
Dietary quality (per 10 KHEI score) ^a^	1.10 (1.00, 1.20)*	1.06 (0.98, 1.15)
Sodium intake (per 100 mg) ^a^	1.00 (0.99, 1.00)	1.00 (1.00, 1.01)
Dietary adherence for hypertension^b^		
Non-adherent	1.00	1.00
Slightly adherent	1.01 (0.80, 1.27)	1.06 (0.87, 1.30)
Highly adherent	1.54 (0.84, 2.81)	0.92 (0.68, 1.23)
Hypertensive adults with antihypertensive drug treatment (*n* = 3776)		
N (total)/% of blood pressure control	1606/74.1	2170/71.0
Dietary quality (per 10 KHEI score)	1.08 (0.98, 1.20)	1.06 (0.97, 1.14)
Sodium intake (per 100 mg)	1.00 (0.99, 1.00)	1.00 (1.00, 1.01)
Dietary adherence for hypertension		
Non-adherent	1.00	1.00
Slightly adherent	1.02 (0.80, 1.29)	1.12 (0.90, 1.39)
Highly adherent	1.62 (0.82, 3.17)	0.94 (0.69, 1.27)
Hypertensive adults without antihypertensive drug treatment *(n = 331)*		
N (total)/% of blood pressure control	173/48.6	158/61.4
Dietary quality (per 10 KHEI score) ^b^	1.28 (0.97, 1.69)	1.19 (0.87, 1.63)
Sodium intake (per 100 mg) ^b^	1.00 (0.99, 1.01)	1.00 (0.98, 1.02)
Dietary adherence for hypertension		
Non-adherent	1.00	1.00
Slightly adherent	1.02 (0.50, 2.05)	0.54 (0.25, 1.17)
Highly adherent	1.07 (0.20, 5.70)	0.83 (0.26, 2.59)

* *p*-value < 0.05

^a^Adjusted for age, duration of hypertension, comorbid status of cardiometabolic diseases such as stroke, myocardial infarction, angina pectoris, diabetes, or dyslipidemia, and family history of hypertension, obesity, smoking, drinking, walking, antihypertensive drug treatment, HEI score, and sodium intake

^b^Dietary adherence was divided into ‘non-adherent’ (> 2400 mg sodium intake and low quartiles (Q1-Q3) of KHEI score), ‘slightly adherent’ (either ≤2400 mg sodium intake or highest quartile (Q4) of KHEI score), and ‘highly adherent’ (≤ 2400 mg sodium intake and highest quartile (Q4) of KHEI score). The OR was adjusted for age, duration of hypertension, comorbid status of cardiometabolic diseases such as stroke, myocardial infarction, angina pectoris, diabetes, or dyslipidemia, and family history of hypertension, obesity, smoking, drinking, and antihypertensive drug treatment

**Fig. 2:**
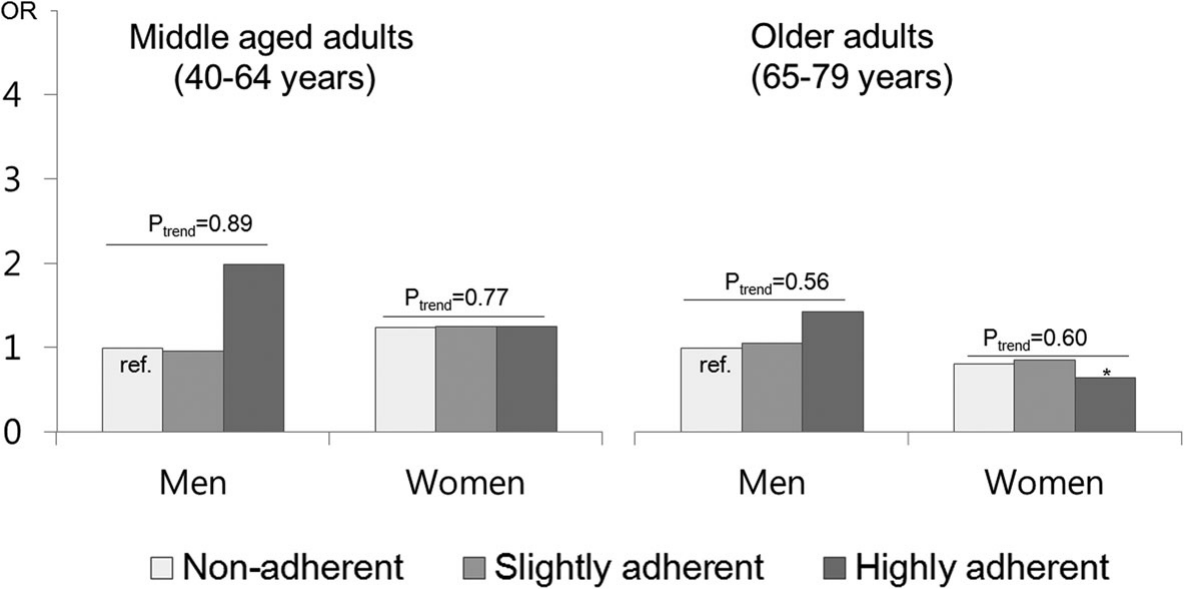
Odds ratio of dietary adherence for blood pressure control by sex and age group among adults with known hypertension. ^1^Odds ratio was adjusted for age, duration of hypertension, comorbid status of cardiometabolic diseases such as stroke, myocardial infarction, angina pectoris, diabetes, or dyslipidemia, and family history of hypertension, obesity, smoking, drinking, walking, and antihypertensive drug treatment. ^2^Dietary adherence was divided into ‘non-adherent’ (> 2400 mg sodium intake and low quartiles (Q1-Q3) of KHEI score), ‘slightly adherent’ (either ≤2400 mg sodium intake or highest quartile (Q4) of KHEI score), and ‘highly adherent’ (≤ 2400 mg sodium intake and highest quartile (Q4) of KHEI score). * < 0.05 of *p*-value for OR for blood pressure control compared with not-adherent men in each age group

**Fig. 3:**
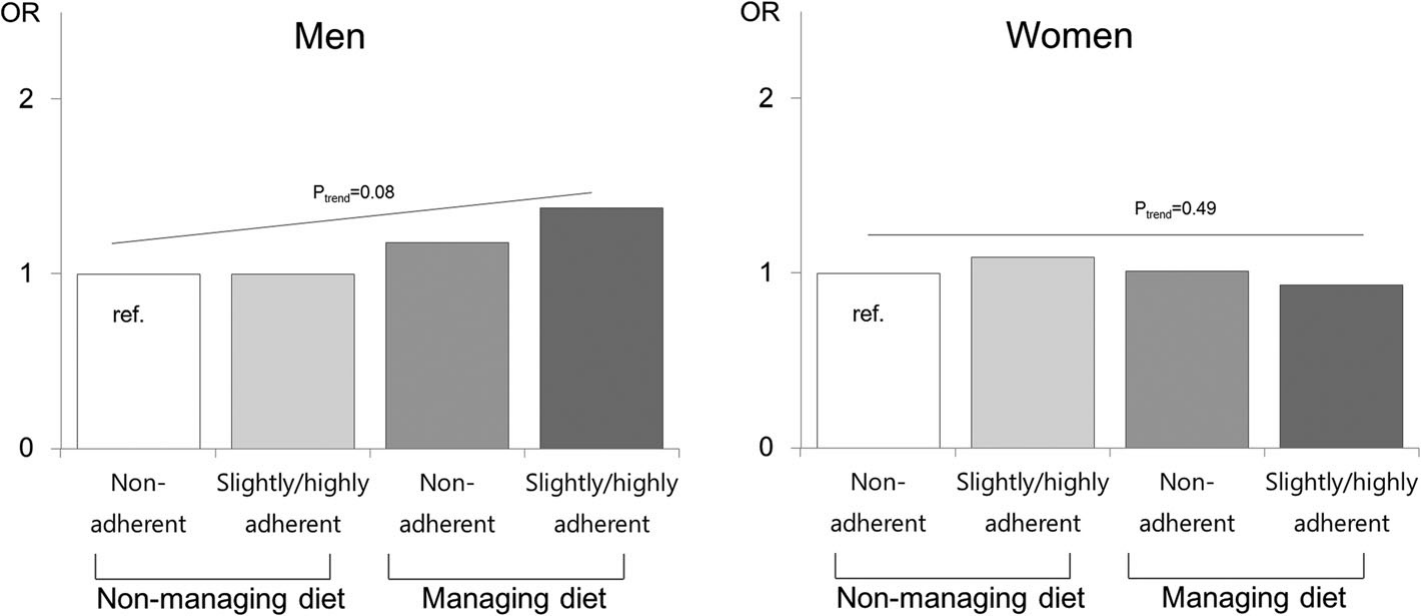
Association of dietary management and adherence for blood pressure control among adults aware of hypertension. ^1^Odds ratio was adjusted for age, duration of hypertension, comorbid status of cardiometabolic diseases such as stroke, myocardial infarction, angina pectoris, diabetes, or dyslipidemia, and family history of hypertension, obesity, smoking, drinking, walking, and antihypertensive drug treatment. ^2^Dietary adherence was divided into ‘non-adherent’ (> 2400 mg sodium intake and low quartiles (Q1-Q3) of KHEI score), ‘slightly adherent’ (either ≤2400 mg sodium intake or highest quartile (Q4) of KHEI score), and ‘highly adherent’ (≤ 2400 mg sodium intake and highest quartile (Q4) of KHEI score)

Figure 2 shows the OR of dietary adherence for BP control according to sex and age group and p for trend across dietary adherence within the same sex and age group. In middle age groups, there was no difference by sex and the level of dietary adherence. However, in older adults, older women with highly adherent diet were more likely to have controlled BP (OR 0.68, 95% CI: 0.44–0.96, *p*-value = 0.02), compared to men with non-adherent diet. We also presented the combined effects of self-reported dietary management practice and dietary adherence on BP control (Fig. 3***)***. Among men, those with higher adherence and self-reported diet management tend to have higher odds for BP control, but the trend did not reach statistical significance (p for trend = 0.08).

## Discussion

This study examined the associations of self-reported diet management practice, dietary quality based-on food and nutrient intakes, adherence to the diet guidelines with BP control among Korean adults with known hypertension. We found that a high-quality diet had a positive association with controlled BP, which was significant only in men.

BP is continuously affected by environmental factors such as diet and physical activity. Prominent effects of diet on BP regulation have been widely identified through numerous epidemiologic studies and clinical trials [4, 5, 7, 23]. A high-quality diet, such as a Mediterranean diet and a diet with a higher HEI score, can prevent development of hypertension in normotensive individuals and can further lower BP in hypertensive individuals already on antihypertensive medication. High-quality diets also reduce cardiovascular risks other than BP [4, 5, 23, 24]. In our study, both diet-managing men and women had higher KHEI scores compared with non-managing individuals and also a higher adherence to the diet recommended for hypertension. Thus, we expected that self-reported diet management practice and high-quality diet would have beneficial effect on BP control. But, the favorable effect of diet was only found in men with borderline significance, and not in women.

In general, women are known to have lower BP levels and higher BP control rates compared with men [25–27]. These results are from the studies of general populations that included normotensive individuals and hypertensive patients together or from studies that included younger adults. In those studies, the higher BP control rate among women was attributed to women’s higher awareness of their health condition, more frequent health service use, more regular medication, a higher therapeutic adherence, and less risky behaviors [25, 26]. However, other studies especially of older populations and/or individuals with hypertension have reported that women’s BP control rates are similar to or lower than men’s [27, 28]. This was consistent with our findings.

According to previous studies, while overall BP levels differ by sex, there was no difference in the benefit of antihypertensive treatment for individuals with hypertension. Also, there was no difference in the impact of dietary modification on BP changes between the sexes according to previous studies [5, 29, 30]. However, several studies have reported sex differences in BP control even when considering potential factors for BP control such as age, co-morbidity, and antihypertensive medication [28, 31]. In a study of older adults with hypertension, women had much higher BP and poorer BP control rates compared with their male counterparts after adjustment for possible factors of BP regulation [28]. Similar to the results of previous studies, older women with known hypertension in our study had a poorer BP control than older men, and no beneficial impact of dietary management practice and dietary quality was found in women. This finding remained unchanged after adjusting for potential confounding variables such as age, comorbidities, health-related behaviors, and BP-lowering drug use. This was also demonstrated in subgroup analysis according to the presence of antihypertensive medication. We cannot definitely explain the lack of a favorable effect of dietary management on BP control in women. Other factors other than age, co-morbidities, obesity, or socio-demographics may explain sex differences in BP control. This was suggested in a previous study [28]. One possible explanation is that women may need a much more aggressive dietary change to obtain a visible dietary impact on BP control. Young women typically have lower BP than young men. However, with advancing years, their BPs are no different from those of men of the same age; and various advantages in women’s BP regulation may decreased with aging. Previous studies suggested that menopausal women, in addition to having sex hormone profile changes, have increased salt sensitivity due to aging compared with men and young women [32, 33]. Thus, a diet much lower in sodium may be more effective in BP reduction in these older women. Although diet-managing women in our study consumed less dietary sodium and much higher-quality diets than non-managing women, the average intake of dietary sodium in both groups was over the recommended amount for hypertensive patients. Hence, dietary quality may not be enough to expect an impact on BP control. Another possible explanation for the sex differences is that health issues due to aging and sex-specific conditions differ between men and women. The magnitude of the negative effects of these issues and conditions may be greater in women. At young age, women generally have fewer unhealthy behaviors compared with men. However, with aging, women have more cardiovascular risks caused by rapid change in body composition, resulting in poor BP control [34]. Moreover, an imbalance between androgens and female sex hormones, especially the loss of estrogen, among postmenopausal women may increase sympathetic activity and adrenergic vasoconstrictor responsiveness, resulting in BP increase [35]. In recent studies, sex-specific effects on T-cell activation and differentiation have been suggested as a potential mechanism in BP regulation [36]. In summary, detrimental changes followed by both chronological and/or ovarian aging of women make BP control difficult. A third possible explanation is the location at which BP was measured. In our study, BP was measured in an office. This may not be sufficient to assess the BP status of women. A previous study suggested that, in women, office-based BP measurements were much higher than 24-h ambulatory measurements [37]. This creates the possibility of underestimation of BP control. Another possible explanation is that diet-managing men in our study had distinctly better health behaviors (less smoking, less drinking, and more walking) compared with non-managing men, while such a tendency was weak in women. In addition, the prevalence of dietary management was lower in women on antihypertensive medication. These findings may result in different associations of diet with BP control between the sexes.

Among the three diet indicators, which were used in this study, only dietary quality was significantly associated with BP control. These results are partly explicable. In our study, the dietary management practice was defined solely on the basis of self-report. Patients who think they are good at dietary management may actually have a poor-quality diet. This suggests that it is necessary to improve the quality of the actual diet, rather than the diet management practice evaluated by oneself.

Our study had several strengths. Use of the KNHANES data ensures that our study includes a number of hypertensive individuals of diverse backgrounds, such as region and socioeconomic status. We could also analyze vast quantities of data to examine self-reported dietary management, quality, and adherence and BP control. To the best of our knowledge, this is the first study to investigate an association between self-reported practices and their quality and BP control in hypertensive individuals.

Several limitations also should be considered. First, the KNHANES is a cross-sectional survey. Thus, our findings do not support a causal relationship between diet and BP control. Second, our study may not have enough statistical power to carry out sex-specific analysis, although the study sample size is not so small. Meaningful interpretations were difficult for some results, because they had only borderline level of statistical significance. For instance, diet-managing men had a higher possibility of having controlled BP than men non-diet-managing men with borderline significance (OR: 1.27, 95% CI: 0.98–1.66, *p*-value = 0.07). Third, we evaluated dietary quality and adherence using single-day 24-h recall data, which might not reflect long-term nutritional intake. Thus, dietary quality and adherence evaluated in our study have a possibility of classification error. To reduce the influence of error, we repeated the analysis only for those who reported that the diet on the recall day was typical. The results remained unchanged (Additional file 1: Table S1 and S2). BP measurement must also be considered. The KNHANES BP measurements were performed in the office according to a strictly predefined protocol. However, differences in office-based versus 24-h ambulatory BP measurements have been demonstrated [37]. The resulting misclassification may have affected the results in our study.

## Conclusions

A high-quality diet was positively associated with BP control in Korean men with known hypertension. Our findings highlight the beneficial impact of dietary management as a means for achieving blood pressure control.

## Additional file

**Additional file 1: Table S1.** Odds ratio of self-reported dietary management for blood pressure control among adults with those who were aware of their hypertension and ate as usual on the recall day. **Table S2.** Odds ratio of dietary quality, sodium intake, and adherence for blood pressure control among those who were aware of their hypertension and ate as usual on the recall day.

## Data Availability

The datasets used and/or analyzed during the current study are available from the corresponding author on reasonable request.
