# Supplementary material for: Self-reported diet management, dietary quality, and blood pressure control in Korean adults with hypertension
Source: Clin Hypertens. 2019 Dec 15;25:24. doi: 10.1186/s40885-019-0130-z (PMC6911701; doi:10.1186/s40885-019-0130-z)
Supplement: Supplementary file 1 — Additional file 1: Table S1. Odds ratio of self-reported dietary management for blood pressure control among adults with those who were aware of their hypertension and ate as usual on the recall day. Table S2. Odds ratio of dietary quality, sodium intake, and adherence for blood pressure control among those who were aware of their hypertension and ate as usual on the recall day. [file 40885_2019_130_MOESM1_ESM.docx]

Supplementary table 1. Odds ratio of self-reported dietary management for blood pressure control among adults with those who were aware of their hypertension and ate as usual on the recall day

|  |  | Non-managing | |  | Managing | |  | OR (95% CI) of dietary management for BP control | | |
| --- | --- | --- | --- | --- | --- | --- | --- | --- | --- | --- |
|  |  | No. of  Total | No. of controlled (%) |  | No. of  Total | No. of controlled (%) |  | Model 1^1^ | Model 2^2^ | Model 3^3^ |
| ***All adults*** ***with known hypertension*** | | | | | |  |  |  |  |  |
| Men |  | 1059 | 746 (70.4) |  | 317 | 242 (76.3) |  | 1.34 (1.00, 1.80)* | 1.30 (0.96, 1.75) | 1.32 (0.97, 1.80) |
| Women |  | 1290 | 906 (70.2) |  | 497 | 3446 (69.6) |  | 0.95 (0.75, 1.19) | 0.97 (0.77, 1.23) | 0.96 (0.76, 1.22) |
| ***Adults with antihypertensive drug treatment*** | | | | | |  |  |  |  |  |
| Men |  | 963 | 694 (72.1) |  | 291 | 230 (79.0) |  | 1.45 (1.06, 1.99)* | 1.47 (1.06, 2.03)* | 1.44 (1.04, 2.02)* |
| Women |  | 1212 | 855 (70.5) |  | 451 | 321 (71.2) |  | 1.00 (0.79, 1.27) | 1.04 (0.81, 1.34) | 1.02 (0.79, 1.31) |
| ***Adults without antihypertensive drug treatment*** | | | | | |  |  |  |  |  |
| Men |  | 96 | 52 (54.2) |  | 26 | 12 (46.2) |  | 0.76 (0.32, 1.84) | 0.81 (0.32, 2.03) | 1.14 (0.40, 3.20) |
| Women |  | 78 | 51 (65.4) |  | 46 | 25 (54.4) |  | 0.63 (0.30, 1.34) | 0.57 (0.26, 1.24) | 0.55 (0.24, 1.27) |

^1^Adjusted for age.

^2^Adjusted for duration of hypertension, comorbid status of cardiometabolic diseases such as stroke, myocardial infarction, angina pectoris, diabetes, or dyslipidemia, and family history of hypertension plus variables in the model 1.

^3^Adjusted for obesity, smoking, drinking, walking, and antihypertensive drug treatment plus variables in the model 2.

*p value <0.05

Supplementary table 2. Odds ratio of dietary quality, sodium intake, and adherence for blood pressure control among those who were aware of their hypertension and ate as usual on the recall day

|  | ORs for blood pressure control | |
| --- | --- | --- |
|  | Men | Women |
| ***All adults with known hypertension (n=3163)*** | | |
| N (total)/% of blood pressure control | 1376 / 71.8 | 1787 / 70.1 |
| Dietary quality (per 10 KHEI score) ^1^ | 1.11 (1.01, 1.24)* | 1.06 (0.97, 1.16) |
| Sodium intake (per 100mg) ^1^ | 1.00 (0.99, 1.00) | 1.00 (1.00, 1.01) |
| Dietary adherence for hypertension^2^ |  |  |
| Non-adherent | 1.00 | 1.00 |
| Slightly adherent | 1.05 (0.81, 1.35) | 1.07 (0.85, 1.35) |
| Highly adherent | 1.54 (0.79, 3.01) | 0.92 (0.66, 1.28) |
| ***Hypertensive adults with antihypertensive drug treatment (n=2917)*** | | |
| N (total)/% of blood pressure control | 1254 / 73.7 | 1663 / 70.7 |
| Dietary quality (per 10 KHEI score) | 1.10 (0.98, 1.23) | 1.06 (0.96, 1.16) |
| Sodium intake (per 100mg) | 1.00 (0.99, 1.00) | 1.00 (1.00, 1.01) |
| Dietary adherence for hypertension |  |  |
| Non-adherent | 1.00 | 1.00 |
| Slightly adherent | 1.05 (0.79, 1.38) | 1.15 (0.90, 1.46) |
| Highly adherent | 1.73 (0.82, 3.65) | 0.94 (0.67, 1.32) |
| ***Hypertensive adults without antihypertensive drug treatment (n=246)*** | | |
| N (total)/% of blood pressure control | 122 / 52.5 | 124 / 61.3 |
| Dietary quality (per 10 KHEI score) ^2^ | 1.26 (0.90, 1.76) | 1.09 (0.74, 1.59) |
| Sodium intake (per 100mg) ^2^ | 1.01 (0.99, 1.02) | 1.00 (0.98, 1.02) |
| Dietary adherence for hypertension |  |  |
| Non-adherent | 1.00 | 1.00 |
| Slightly adherent | 1.05 (0.45, 2.44) | 0.53 (0.23, 1.25) |
| Highly adherent | 0.56 (0.07, 4.46) | 0.89 (0.23, 3.48) |

^1^Adjusted for age, duration of hypertension, comorbid status of cardiometabolic diseases such as stroke, myocardial infarction, angina pectoris, diabetes, or dyslipidemia, and family history of hypertension, obesity, smoking, drinking, walking, antihypertensive drug treatment, HEI score, and sodium intake.

^2^Dietary adherence was divided into ‘non-adherent’ (>2400mg sodium intake and low quartiles (Q1-Q3) of KHEI score), ‘slightly adherent’ (either ≤ 2400mg sodium intake or highest quartile (Q4) of KHEI score), and ‘highly adherent’ (≤ 2400mg sodium intake and highest quartile (Q4) of KHEI score). The OR was adjusted for age, duration of hypertension, comorbid status of cardiometabolic diseases such as stroke, myocardial infarction, angina pectoris, diabetes, or dyslipidemia, and family history of hypertension, obesity, smoking, drinking, and antihypertensive drug treatment.

*p value <0.05
